# Remote Regulation of Molecular Diffusion in Extracellular Space of Parkinson’s Disease Rat Model by Subthalamic Nucleus Deep Brain Stimulation

**DOI:** 10.34133/cbsystems.0218

**Published:** 2025-04-03

**Authors:** Dan Du, Wanyi Fu, Shaoyi Su, Xin Mao, Liu Yang, Meng Xu, Yi Yuan, Yajuan Gao, Ziyao Geng, Yanjing Chen, Mingming Zhao, Yu Fu, Feng Yin, Hongbin Han

**Affiliations:** ^1^ Department of Radiology, Peking University Third Hospital, Beijing 100191, China.; ^2^ Department of Magnetic Resonance Imaging, Qinhuangdao Municipal No. 1 Hospital, Qinhuangdao 066000, China.; ^3^ Department of Electronic Engineering, Tsinghua University, Beijing 100084, China.; ^4^ Beijing Key Laboratory of Magnetic Resonance Imaging Devices and Technology, Peking University Third Hospital, Beijing 100191, China.; ^5^ Institute of Medical Technology, Peking University Health Science Center, Beijing 100191, China.; ^6^ School of Electrical Engineering, Yanshan University, Qinhuangdao 066004, China.; ^7^ National Medical Products Administration Key Laboratory for Evaluation of Medical Imaging Equipment and Technique, Beijing 100191, China.; ^8^ Department of Neurosurgery, Aerospace Center Hospital, Beijing 100049, China.; ^9^ Department of Neurology, Peking University Third Hospital, Beijing 100191, China.

## Abstract

Subthalamic nucleus deep brain stimulation (STN-DBS) is an effective therapy for Parkinson’s disease (PD). However, the therapeutic mechanisms remain incompletely understood, particularly regarding the extracellular space (ECS), a critical microenvironment where molecular diffusion and interstitial fluid (ISF) dynamics are essential for neural function. This study aims to explore the regulatory mechanisms of the ECS in the substantia nigra (SN) of PD rats following STN-DBS. To evaluate whether STN-DBS can modulate ECS diffusion and drainage, we conducted quantitative measurements using a tracer-based magnetic resonance imaging. Our findings indicated that, compared to the PD group, STN-DBS treatment resulted in a decreased diffusion coefficient (D*), shorted half-life (*T*_1/2_), and increased clearance coefficient (*k*′) in the SN. To investigate the mechanisms underlying these changes in molecular diffusion, we employed enzyme-linked immunosorbent assay (ELISA), Western blotting (WB), and microdialysis techniques. The results revealed that STN-DBS led to an increase in hyaluronic acid content, elevated expression of excitatory amino acid transporter 2 (EAAT2), and a reduction in extracellular glutamate concentration. Additionally, to further elucidate the mechanisms influencing ISF drainage, we employed immunofluorescence and immunohistochemical techniques for staining aquaporin-4 (AQP-4) and α-synuclein. The results demonstrated that STN-DBS restored the expression of AQP-4 while decreasing the expression of α-synuclein. In conclusion, our findings suggest that STN-DBS improves PD symptoms by modifying the ECS and enhancing ISF drainage in the SN regions. These results offer new insights into the mechanisms and long-term outcomes of DBS in ECS, paving the way for precision therapies.

## Introduction

Deep brain stimulation (DBS) is a neuromodulation therapy [1] that has proven effective in treating medication-refractory neurological disorders like tremors, rigidity, and epilepsy, especially in Parkinson’s disease (PD) patients. Since its approval by the U.S. Food and Drug Administration (FDA), DBS has been applied to over 200,000 patients globally, with numbers drastically increasing [2]. Subthalamic nucleus DBS (STN-DBS) has emerged as a frontline treatment to alleviate PD symptom [3,4]. Since the initial research by Benazzouz et al. [5] demonstrated that high-frequency STN stimulation enhances motor function by inactivating local neurons and dampening excessive STN activity, substantial progress has been made in clinical application and mechanism understanding of DBS. Current research studies usually focus on neuronal depolarization [6,7], neurotransmitter roles [8], neuroinflammation [9,10], and neural circuitry [11], yet clinical inconsistencies in treatment efficacy and duration [12] suggest that neuron-centric models fall short in explaining therapeutic outcomes fully.

Emerging research underscores the extracellular space (ECS) [13,14], ranging from 20 to 60 nm, as the most direct living space for neurons—the microenvironment directly surrounding neurons, composed of the extracellular matrix (ECM) and interstitial fluid (ISF)—as a pivotal factor in PD progression. The ECS functions as a microcirculatory system for ISF, essential for neural excitability and substance transportation and drainage. In PD, ECM alterations lead to degradation [15,16], inflammation, and the release of molecules [17] like hyaluronic acid (HA) fragments, tenascin, and sulfated proteoglycans, which act as paracrine signals and potential biomarkers for early diagnosis and PD monitoring [18]. Moreover, impaired ISF drainage is linked to Aβ accumulation and neuronal degeneration [19,20]. Glymphatic system dysfunction [21–23] results in heightened α-synuclein (α-syn) aggregation and dopaminergic neuron loss in PD rat models [24]. While in vitro studies indicate that DBS can stimulate ECM protein production [25], enhancing intercellular communication, these findings await in vivo validation. Notably, the impact of DBS on ECS, particularly ECM modulation and ISF drainage, remains unexplored, highlighting the ECS as a key area for advancing DBS mechanisms in PD treatment.

To investigate this cellular microenvironment, techniques such as real-time ion measurement [26], integrated optical imaging, fluorescence recovery after photobleaching, and diffusion-weighted magnetic resonance imaging (MRI) have been employed [27]. These methods have revealed ECS properties and functions. However, they usually focus on shallow detection depths, typically restricted to areas within 200 μm of brain tissue [28], and offer a single ECS indicator, namely, the volume fraction, which reflects the indicator of ECS structure. To capture dynamic ECS probe concentrations, our laboratory previously developed tracer-based MRI [29–32] and image processing to reveal 3-dimensional (3D) imaging of brain microenvironment fluid dynamics in real time [33]. This work demonstrated that PD rat models exhibit increased tracer half-life (*T*_1/2_) and decreased clearance coefficient (*k*′) of ECS in substantia nigra (SN) regions, indicative of impaired transport and detoxification [34–36]. However, whether DBS can regulate the changes of ECS in the SN remains to be explored.

This study evaluates how STN-DBS modulates ECM components and ISF drainage in PD rat models, aiming to understand its remote effects in the SN. We conducted quantitative analyses of ECS parameters before and after STN-DBS and employed microdialysis, enzyme-linked immunosorbent assay (ELISA), and immunohistochemistry to assess extracellular levels of glutamate, HA, and α-syn. These analyses aim to clarify molecular transport mechanisms within the ECS and alterations in ISF drainage. Furthermore, we performed behavioral assessments, electroencephalography, and tyrosine hydroxylase (TH) staining to gauge therapeutic efficacy and neuroprotection. This study provides insights into neuroprotective mechanisms of DBS in a PD rat model, laying the theoretical groundwork for novel treatment approaches.

## Materials and Methods

### Experimental design

**Fig. 1.**
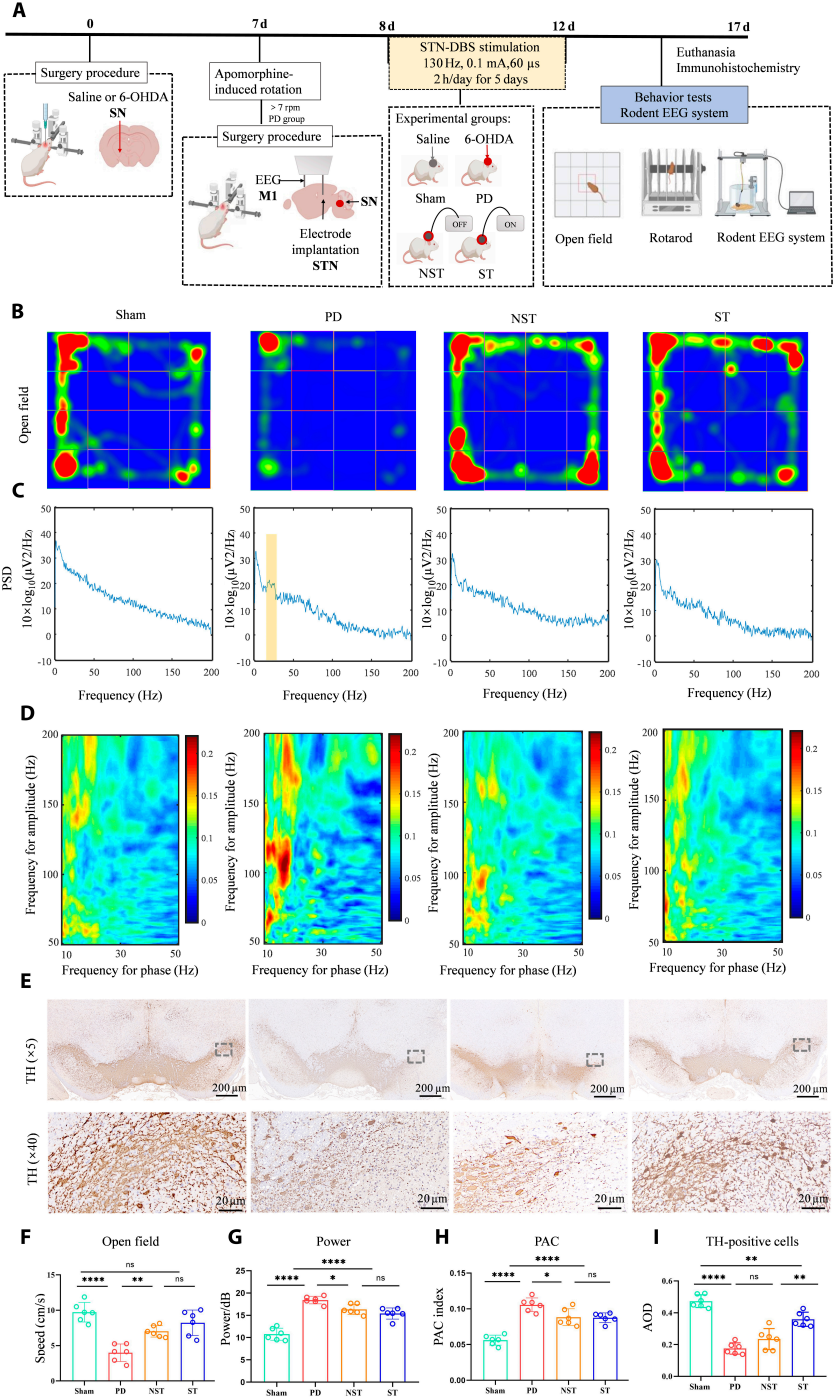
Experimental design and protective effects of STN-DBS across different groups. (A) Experimental design. (B) Behavior assessments using open field tests. Heatmaps illustrate time spent (red) and movement trajectories (green). The PD group exhibited decreased movement speed compared to the Sham group; however, both the NST and ST groups showed increased movement speed relative to the PD group. (C) EEG signals from the right M1. The PD group exhibited a typical β band, while EEG patterns in the NST and ST groups were similar to those in the Sham group. (D) Phase-amplitude coupling (PAC) analysis in the right M1. The PD group exhibited beta/ripple coupling, absent in the NST and ST groups. (E) TH staining in the right SN. TH-positive cell counts in the PD group were lower than the Sham group. The NST group showed levels comparable to the PD group, while the ST group had more TH-positive cells than both the PD and NST groups. The square represents the target area in the right SN where 6-OHDA was injected. (F to I) Boxplots show (F) the speed in the open-field test, (G) EEG power, (H) PAC, and (I) TH-positive cell counts. Circles represent individual values, and error bars show SD. Statistical analysis was conducted using one-way ANOVA followed by FDR correction. *P < 0.05, **P < 0.01, ****P < 0.0001, and ns indicating no significant difference. Figure created with BioRender.com.

**Fig. 2.**
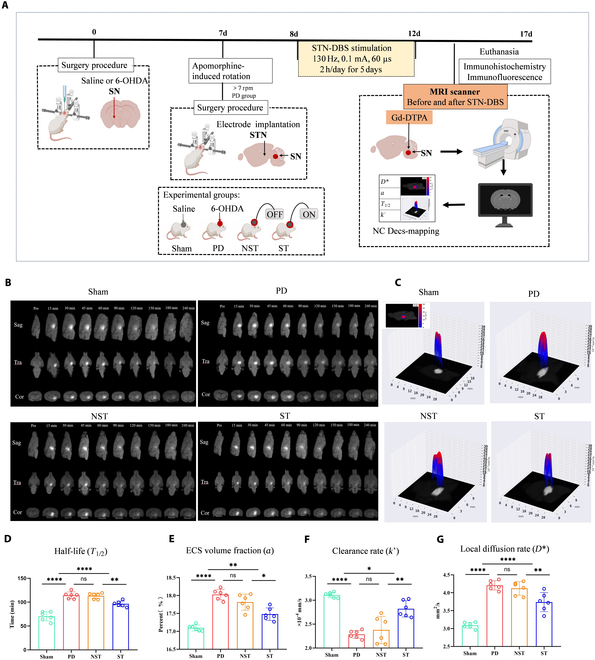
(A) Flowchart of experimental design for measuring ECS parameters via MRI. Five days after stimulation, a paramagnetic probe gadolinium diethylenetriaminepentaacetic acid (Gd-DTPA) was injected into the right SN of each rat. MRIs were scanned at designated time points. The MR images were analyzed to derive ECS parameters, followed by euthanasia and brain collection. (B) MR images with the tracer in the ECS (high signal intensity) are shown in coronal, axial, and sagittal planes. In the Sham group, high signal areas largely disappeared after 240 min, similar to the ST group. The PD group retained tracer residues, similar to the NST group. (C) 3D Decs-mapping diagrams show the diffusion coefficient (D*) contour lines in the right SN. Peak values of D* of the PD and NST groups were higher than those of the Sham and ST groups. (D to G) Boxplots of ECS structure and ISF drainage parameters in the right SN. The PD and NST groups showed longer *T*_1/2_, increased *α*, reduced *k*′, and higher D* compared to the Sham group, with no significant differences between PD and NST. In contrast, the ST group exhibited significant improvements for these parameters. Circles represent individual values, and error bars represent SD. One-way ANOVA was performed followed by FDR correction. *P < 0.05, **P < 0.01, ****P < 0.0001, and ns indicates no significant difference.

Under stereotactic conditions, Sprague–Dawley rats were included in this study and randomly assigned to (a) Sham group (*n* = 6), receiving saline injection into the right SN; (b) PD group (*n* = 6), receiving 6-hydroxydopamine (6-OHDA) injection into the right SN as a PD model; (c) NST group (*n* = 6), receiving 6-OHDA injection into the right SN with electrode implantation but no stimulation; and (d) ST group (*n* = 6), receiving 6-OHDA injection into the right SN with electrode implantation and stimulation. To confirm the PD model, 1 week after the SN injection, the rats were assessed with an apomorphine-induced rotation test with a constant speed of 7 rpm for a duration of 30 min. On the 8th day, stimulation was applied to the ST group for 5 consecutive days and ceased on the 12th day. The next day after the procedure, all rats were evaluated with (a) behavioral experiments, including open-field test and rotarod test (Fig. 1A), (b) electroencephalographic recordings from the primary motor cortex (M1) of the right cerebral hemisphere (Fig. 1A), and (c) tracer-based MRI scans (Fig. 2A). After the final scan, the animals were euthanized for immunohistochemical and immunofluorescence analyses.

**Fig. 3.**
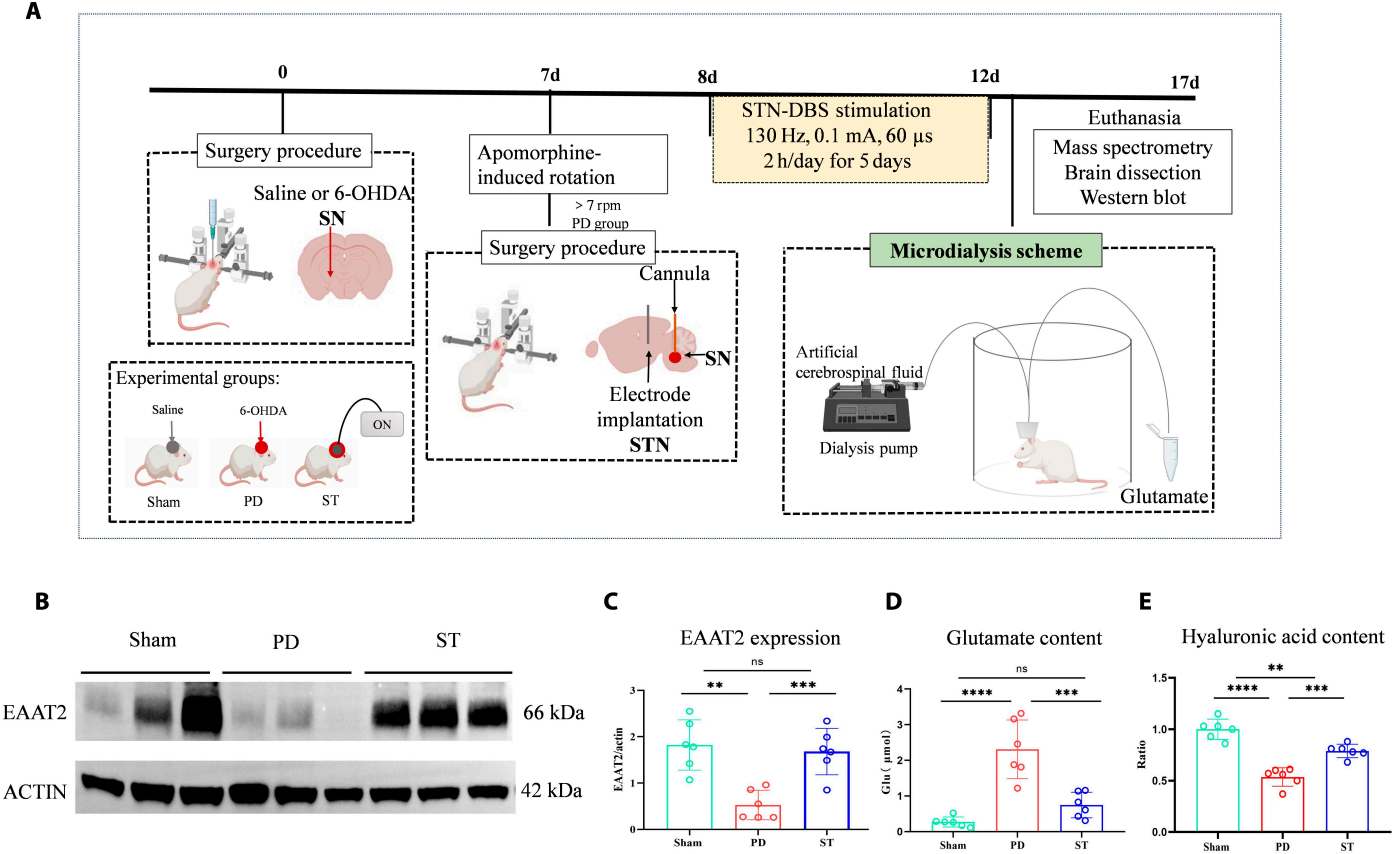
(A) Illustration of the microdialysis process. After STN-DBS treatment, extracellular neurotransmitters were collected from the SN on the injured side of each group of rats while they were awake. At the end of the experiment, mass spectrometry analysis, brain slice preparation, and Western blot experiments were conducted. (B and C) WB findings for each group. In comparison to the Sham group, EAAT2 expression was diminished in the PD group but elevated following STN-DBS. (D) Quantitative examination of extracellular glutamate revealed that, relative to the Sham group, the glutamate concentration in the ECS was notably higher in the PD group. However, after STN-DBS treatment, the extracellular glutamate level declined in the ST group. (E) Variations in HA content across groups. When compared to the Sham group, HA content was significantly reduced in the PD group, whereas it was augmented in the ST group. Circles represent individual values, and error bars represent SD. One-way ANOVA was performed followed by FDR correction. **P < 0.01, ***P < 0.001, ****P < 0.0001, and ns indicates no significant difference.

Furthermore, to monitor extracellular glutamate levels, following the aforementioned surgical interventions, an additional cohort of animals received cannulas implanted in the right SN. These animals were allocated into 3 groups: Sham (*n* = 6), PD (*n* = 6), and ST (*n* = 6). Microdialysis was conducted on these animals prior to and following both the initial and final DBS sessions, enabling the assessment of glutamate concentrations released in the right SN through liquid chromatography–mass spectrometry methods (Fig. 3A).

### Experimental animals

A total of 47 male Sprague–Dawley rats, weighing between 230 and 250 g, were provided by the Department of Animal Sciences at Peking University Health Science Center. The study excluded 5 rats due to premature death or issues with the implantation procedure. The experimental design was approved by the Ethics Committee of Aerospace Center Hospital (ethics agreement no. 2021-NSFC-004). The rats were maintained in an environment with a temperature of 22 ± 1 °C and a relative humidity of 60 ± 5%, under a 12-h light–dark cycle. They were provided with food and water without restrictions.

### Surgical procedure for PD model

The rats were subjected to anesthesia with 4% isoflurane (RWD Life Science, catalog no.: R510-22-10) and were positioned in a stereotaxic apparatus (Stoelting Co., Illinois, USA), with the anesthesia level maintained at 1.5% during the surgery. The scalp was surgically opened along the midline to reveal the bregma. A microinjection syringe (Hamilton Bonaduz AG, Bonaduz, Switzerland) was employed to deliver 4 μl of 6-OHDA solution (Sigma Chemical Co., St. Louis, MO, USA; 7.5 μg/μl saline with the addition of 0.2% ascorbic acid) or an equivalent volume of saline (with 0.2% ascorbic acid) into the right SN, using the following coordinates in relation to the bregma: anterior-posterior (AP), −4.8 mm; medial-lateral (ML), −2.0 mm; dorsal-ventral (DV), +7.8 mm. The infusion rate was controlled at 0.4 μl/min. After the administration, the needle was left in place for an additional 5 min before it was carefully withdrawn. The incision was sutured, and prophylactic antibiotics were administered to minimize the risk of infection.

### Electrode implantation procedure

Seven days after the injection of 6-OHDA into the right SN, a customized electrode (CWMA-S12S electrode, consisting of a 12-channel recording electrode and a 2-channel stimulation electrode, manufactured by Blackrock) was implanted into the right STN (coordinates: AP, −2.5 mm; ML, −3.7 mm; DV, +7.5 mm) and the right M1 area (coordinates: AP, −2.0 mm; ML, −1.6 mm; DV, +1.0 mm). A screw was implanted into the parietal cortex above the contralateral skull (coordinates: AP, −6.0 mm; ML, +1.6 mm; DV, +1.0 mm) to serve as the anode. The electrode was secured to the skull using dental acrylic cement to fix the screw. After electrode implantation, the subjects were administered nonsteroidal anti-inflammatory drugs and penicillin.

### DBS protocol and electrophysiological signal acquisition

On the 8th day after 6-OHDA injection, the ST group received DBS treatment using a stimulator (YC-3B dual-channel programmable stimulator, model B205, Chengdu Instrument Factory, China) with parameters set at 130 Hz, 60-μs pulse width, 0.1 mA, for 2 h per day. STN-DBS was performed for 5 consecutive days from 9:00 AM to 11:00 AM.

Local field potentials (LFPs) from the right M1 were recorded using the Cereplex Direct system (Blackrock, USA). The parameters were set at band-pass (BP) of 250 Hz to 5 kHz, low-pass (LP) of 500 Hz, and a sampling rate of 2 kHz. The Welch method and phase locking value algorithm were employed to calculate the average power spectral density and phase-amplitude coupling (PAC), respectively. A digital amplifier (Cereplex μ) was used to acquire electroencephalogram (EEG) and LFP signals.

### Behavioral evaluation

#### Apomorphine-induced rotation test

To validate the PD model, 7 d after injection, animals were administered 0.05% apomorphine (0.5 mg/kg) via intraperitoneal injection [37,38]. The rats were allowed to acclimate in a quiet, isolated room for 5 min before recording the number of contralateral rotations for each animal over 30 min. A rotation speed greater than 7 rpm indicated the successful establishment of the PD model [34].

#### Rotarod test

After the pre-experimental phase, every animal underwent training on the rotarod at a steady, low speed of 5 rpm for a duration of 5 min. The experiment was initiated after a 1-h interval. The parameters were set as follows: an initial speed of 4 rpm for 10 s, followed by gradual acceleration over 100 s to a maximum speed of 40 rpm, which was maintained for 180 s. The average fall time was selected as an indicator of motor ability. Each rat was subjected to the test twice, and the results were averaged. Additionally, the equipment was thoroughly cleaned and disinfected between each test for every individual rat.

#### Open-field test

An open-field test was conducted using a black square arena with dimensions 100 cm × 100 cm × 40 cm. A camera was fixed directly above the arena for 10 min to record the entire spontaneous activity within the arena. The experimental arena was cleaned and disinfected before and after each test. Activity parameters, including movement distance, movement speed, trajectory maps, and heat maps, were obtained using the Visu Track animal behavior analysis software.

### Method and parameter calculation based on MRI

A specifically distributed paramagnetic probe was introduced in the right SN of the experimental animals. In a magnetic field environment, the probe alters the longitudinal relaxation time of hydrogen nuclei in water molecules, which manifests as a high signal in MR images. This allows for the modeling, processing, and analysis of changes in the physical signals detected before and after probe introduction, thereby solving the structural and functional characteristic parameters of the ECS. To describe the distribution and transport patterns of the probe within the ECS after its introduction, this system primarily establishes a 3D anisotropic diffusion modeling equation, as shown in Eq. (1):

$$\frac{\partial C}{\partial t}=D_{x}\frac{\partial^{2}C}{\partial x^{2}}+D_{y}\frac{\partial^{2}C}{\partial y^{2}}+D_{z}\frac{\partial^{2}c}{\partial z^{2}}-\mathrm{kC}+\frac{Q}{\alpha }$$ (1)

where *Q* signifies the signal source that is injected, while *C* indicates the concentration of the probe at a given time *t* and spatial coordinates (*x*, *y*, *z*). *D_x_*, *D_y_*, and *D_z_* symbolize the diffusion coefficients corresponding to the *x*, *y*, and *z* directions, respectively. *k* is associated with the clearance coefficient linked to tracer metabolism within the body, and *a* represents the proportion of the ECS volume in brain tissue. Detailed procedures for modeling and solving this model can be found in the literature [39]. Subsequently, the ADCe file, a quantifiable image file generated after calculating the diffusion coefficient of the microstructure of the rat brain ECS from the acquired MR images, was selected from the processed image directory, and 2D and 3D wireframe Decs-mapping visualization images were drawn using the Python package matplotlib. The dataset was formatted with the “lowest value” set to blue and the “highest value” set to red. In the 3D graph, the height represents the signal intensity of the diffusion parameter Decs in the MR image, and the *XY* screen at the bottom of the graph is a contour plot superimposed on the original MR image.

The ECS structural parameters and ISF diffusion indices, which encompass the volume fraction (*α*), diffusion coefficient (D*), clearance coefficient (*k*′), and half-life (*T*_1/2_), were ascertained employing an MRI-based tracer methodology. For the assessment, 6 rats from each group were selected at random before and following STN-DBS stimulation, which occurred at 7 to 12 d and 17 to 22 d after injection. A direct injection of 2 μl of a 10 mM magnetic tracer solution, gadolinium-diethylene triamine pentaacetic acid (Bayer Schering Pharma AG, Berlin, Germany), was administered into the SN (relative to the bregma: AP, −4.8 mm; ML, + 2.0 mm; DV, −7.8 mm).

Brain imaging of the rats was conducted using a 3.0-T MRI scanner (Magnetom Trio, Siemens Medical Solutions, Erlangen, Germany) equipped with an 8-channel coil. T1-weighted 3D magnetization-prepared rapid acquisition gradient echo sequences were utilized to capture the images, with the sequence parameters adapted from Han’s research [23]. The acquisition parameters are as follows: echo time = 3.7 ms, repetition time = 1,500 ms, flip angle = 12°, inversion time (TI) = 900 ms, field of view = 267 mm, voxel = 0.5 mm × 0.5 mm × 0.5 mm, matrix = 512 × 512, average = 2, and phase encoding steps = 96. The imaging session for each rat lasted 290 s. In this study, the ECS parameter calculations were executed using an in-house developed brain ECS measurement software, the Nano-Detect Analyze system V1.2. The steps followed the optimized free diffusion equation for the solution, as described previously by our research group [40].

### Immunohistochemistry of TH

Damage to the dopaminergic neurons was assessed by measuring the expression of TH protein in the right SN. After the experiment, rats in each group were anesthetized by abdominal injection and euthanized by decapitation for brain extraction. Coronal sections were obtained from each brain and subsequently sliced into 4-μm-thick sections traversing the ventral midbrain. These sections underwent overnight incubation at 4 °C with anti-TH antibody (1:1,000; GB11181; Servicebio), followed by a 50-min incubation at room temperature with horseradish peroxidase-labeled goat anti-rabbit immunoglobulin G (IgG) (1:200; GB23303; Servicebio). The sections were ultimately counterstained with hematoxylin and eosin, and the presence of TH protein in the SN of the hemisphere that received the 6-OHDA injection was examined using an optical microscope (model E100; Nikon).

### Microdialysis procedure

A microdialysis probe was inserted into the right SN of rats, followed by perfusion with artificial cerebrospinal fluid at a flow rate of 1 μl/min for 1 h to equilibrate, alleviate tissue trauma caused by implantation and potential rejection reactions in the rat body, and observe the rats’ vital signs. After the vital signs of the rats stabilized, the sampling interval was adjusted to 60 min, and samples were collected continuously for 240 min. Each collected sample was promptly stored in a −80 °C freezer. In the ST group, intracerebral microdialysis was performed before and after the first and fifth STN-DBS sessions and 12 and 17 d after neurotoxin injection into the SN. The Sham and PD groups received intracerebral microdialysis at the same location and during the same period as the implanted cannulas. This procedure was performed according to a previously established protocol.

The levels of glutamate in the right SN of PD rats were determined by employing liquid chromatography–mass spectrometry. A sample volume of 50 μl was extracted, combined with 150 μl of methanol, mixed via vortexing for 5 min, and then subjected to centrifugation at 13,000 rpm for 10 min. The resulting supernatant was reserved for subsequent analysis.

The liquid chromatography–mass spectrometry analytical conditions were configured as follows: The separation was achieved using an Ultimate XB-C18 column (5 μm, 4.6 mm × 150 mm, Welch); the mobile phase was a mixture of solvent A (water with 0.1% formic acid) and solvent B (acetonitrile with 0.1% formic acid), which was applied with a gradient elution program at a flow rate of 0.3 ml/min; the column was maintained at a temperature of 40 °C; the sample tray was cooled to 4 °C; and the sample injection volume was set at 5 μl.

For the mass spectrometric analysis, the conditions were optimized as follows: An electrospray ionization source was used with a spray voltage of +4,000 V, a nebulization temperature of 350 °C, sheath gas (N2) flow rate of 65 Arb, auxiliary gas (N2) flow rate of 20 Arb, and a collision gas of high-purity nitrogen. The analysis was conducted in the selected reaction-monitoring mode for positive-ion detection. Data acquisition and processing of the chromatograms were managed using the Xcalibur software (Thermo Fisher Scientific). Quantitative analysis was performed using linear regression analysis with a weighting coefficient of 1/X2.

### Western blot

The SN tissue was freshly extracted and carefully homogenized in radioimmunoprecipitation assay (RIPA) buffer (G2002-100ML, Servicebio) at a cold temperature of 4 °C (containing protease inhibitors, with a ratio of RIPA:cocktail:phenylmethylsulfonyl fluoride:phosphatase inhibitor A: phosphatase inhibitor B = 100:2:1:1:1). The Bradford method (Bio-Rad, Hercules, CA, USA) was applied to quantify the protein content. Following protein quantification, the samples were adjusted to the appropriate concentration, and sodium dodecyl sulfate–polyacrylamide gel electrophoresis (SDS-PAGE) was conducted to fractionate the proteins. Once the proteins were separated, they were transferred to a polyvinylidene difluoride (PVDF) membrane (0.45 μm, G6015-0.45, Servicebio), which was pre-activated with ethanol for 2 min. The PVDF membrane, containing the transferred proteins, was placed in an incubation chamber with tris-buffered saline and Tween 20 (TBST), to which 5 ml of a 5% nonfat milk solution was added. The membrane was then set on a rocking platform and allowed to block at ambient temperature for 30 min. After blocking, the membrane was incubated with a rabbit anti-EAAT2 primary antibody overnight at 4 °C (1:500, 22515-1-AP, Proteintech) and a rabbit anti-actin primary antibody (1:1,000, GB11001, Servicebio). This was followed by a 30-min incubation with the corresponding horseradish peroxidase-conjugated secondary antibody (1:3,000, GB23303, Servicebio). The immunoreactive bands were visualized using a chemiluminescent ECL detection kit (G2161-200ML, Servicebio), and the developed images were analyzed with ImageJ software (National Institutes of Health, Bethesda, MD, USA) to quantify the protein expression levels.

### AQP-4 and GFAP immunofluorescence staining

A blocking solution was used to block the sections at room temperature for 30 min. A mixture of aquaporin-4 (AQP-4)-specific antibody (GB12529, Servicebio, 1:500) and glial fibrillary acidic protein (GFAP)-specific antibody (GB11096, Servicebio, 1:500) was prepared and applied to the samples, followed by an overnight incubation at 4 °C. Subsequently, the samples were incubated with labeled goat anti-mouse IgG (GB21301, Servicebio, 1:300) and goat anti-rabbit IgG (GB25303, Servicebio, 1:400) at room temperature in the dark for 1 h. The slides were mounted, and images were captured under appropriate excitation filters using a fluorescence microscope (NIKON ECLIPSE C1, Nikon, Japan). Green fluorescence represents GFAP, and red fluorescence represents AQP-4.

### α-Synuclein immunohistochemistry

The clearance of harmful substances was assessed before and after DBS stimulation by measuring the expression of α-syn. After the experiment, the rats in each group were anesthetized via abdominal injection and sacrificed by decapitation for brain removal. Brains were sectioned coronally and further processed into 4-μm sections through the ventral midbrain region. These slices were incubated with an anti-α-syn antibody (1:1,000; GB11773, Servicebio) at 4 °C for an extended period, followed by a 50-min incubation at room temperature with horseradish peroxidase-conjugated goat anti-rabbit IgG (1:200; GB23303, Servicebio). The sections were then stained with hematoxylin and eosin, and the presence of α-syn in the SN of the hemisphere that had been injected with 6-OHDA was examined under a light microscope (E100, Nikon).

### Statistical analysis

All statistical analyses were performed using GraphPad Prism 8.0 and SPSS 20.0. Data are presented as mean ± SD. Multiple group data comparisons were performed using one-way analysis of variance (ANOVA) with false discovery rate (FDR) correction. Using Pearson correlation analysis, we assessed the relationships between EAAT2 expression, extracellular glutamate levels, HA content, AQP-4 expression, and the structural parameters of the ECS as well as the diffusion indices of ISF (*α*, D*, *k*′, *T*_1/2_), in both the PD and ST groups. Statistical significance was set at *P* < 0.05.

## Results

### Changes in motor function and characteristic EEG in the impaired brain regions of PD rats after STN-DBS treatment

The open-field test (Fig. 1B and F) revealed that, in comparison to the Sham group, the rats in the PD group exhibited a notably decreased average movement speed (*P* < 0.0001). Conversely, both the NST and ST groups demonstrated higher average movement speeds than the PD group (*P* = 0.0009 and *P* < 0.0001). Additionally, no statistically significant difference existed between the NST and ST groups (*P* = 0.1348).

The rotarod test results (Fig. S1) showed that, compared with the Sham group, the average fall time of rats in the PD group was significantly reduced (*P* < 0.0001); the NST group and ST group had longer average fall times than the PD group (*P* < 0.0001, *P* < 0.0001), and there was no statistical difference between the NST group and ST group (*P* = 0.0402).

EEG results from M1 of the right cerebral hemisphere showed that (Fig. 1C and D), compared with the Sham group, the PD group exhibited significant β-band activity, with increased power and PAC (*P* < 0.0001, *P* < 0.0001); the NST group and the ST group had reduced β-band power and PAC compared with the PD group (*P* = 0.0053, 0.0002; *P* = 0.0034, 0.0024), and no statistical difference existed between the NST group and the ST group (*P* = 0.1912, 0.8764) (Fig. 1G and H).

### Protection of dopaminergic neurons in the impaired brain regions of PD rats after STN-DBS treatment

The TH staining outcomes revealed a markedly decreased count of TH-immunoreactive cells in the PD group when compared to the Sham group, with a statistically significant difference (*P* < 0.0001). Interestingly, the NST group showed a similar number of TH-immunoreactive cells to that of the PD group, indicating no significant difference (*P* = 0.0451). In contrast, the ST group demonstrated a significantly higher count of TH-immunoreactive cells compared to both the PD and NST groups (*P* < 0.0001, *P* = 0.0002) (Fig. 1E and I). The protective effects of STN-DBS on behavioral assessments, EEG, and TH staining across different groups were shown in Fig. 1 and Table 1.

**Table 1.** Protective effects of STN-DBS on behavioral assessments, EEG, and TH staining across different groups

	Behavioral evaluation		EEG		Immunohistochemistry
	Fall time (s)	Movement speed (cm/s)	Power	PAC	TH
Sham group	105.5 ± 8.36	9.72 ± 1.38	10.77 ± 1.35	0.056 ± 0.007	0.47 ± 0.04
PD group	41.62 ± 9.18	3.99 ± 1.23	18.42 ± 0.85	0.106 ± 0.009	0.17 ± 0.03
NST group	82.37 ± 5.87	7.02 ± 0.81	16.32 ± 1.14	0.088 ± 0.012	0.23 ± 0.06
ST group	91.57 ± 4.68	8.23 ± 1.79	15.41 ± 1.24	0.087 ± 0.007	0.35 ± 0.04
*P*1	<0.0001	<0.0001	<0.0001	<0.0001	<0.0001
*P*2	<0.0001	0.024	<0.0001	<0.0001	<0.0001
*P*3	<0.0001	0.0009	0.0053	0.0034	0.0451
*P*4	<0.0001	<0.0001	0.0002	0.0024	<0.0001
*P*5	0.0034	0.0693	<0.0001	<0.0001	0.0005

Data are presented as mean ± SD. Multiple group data comparisons were performed using one-way ANOVA with FDR correction. Statistical significance was set at *P* < 0.05. *P*1, Sham group versus PD group; *P*2, Sham group versus NST group; *P*3, NST group versus PD group; *P*4, ST group versus PD group; *P*5, Sham group versus ST group. PD, Parkinson’s disease; NST, electrode implantation but no stimulation; ST, electrode implantation and stimulation; PAC, phase-amplitude coupling; TH, tyrosine hydroxylase.

### Changes in ECS parameters in the impaired brain regions of PD rats after STN-DBS treatment

Drainage and diffusion parameters related to the extracellular microenvironment were obtained using an MRI tracer and Decs-mapping. MR images showed that the tracer had a high signal intensity, and 240 min after tracer injection, the tracer in the right SN of the Sham group was cleared. At the same time point, there were more tracer residues in the PD and NST groups, whereas the tracer in the ST group was cleared (Fig. 2B). Quantitative analysis using Nano-Detect Analysis System software showed that, compared with the Sham group, the impaired brain regions of the PD group had increased D* (*P* < 0.0001), prolonged *T*_1/2_ (*P* < 0.0001), decreased *k*′ (*P* < 0.0001), and increased *a* (*P* < 0.0001). The NST group had similar results to the PD group, with no statistical difference in various parameters (D*, *P* = 0.4247; *T*_1/2_, *P* = 0.7725; *k*′, *P* = 0.3623; *α*, *P* = 0.0382). After STN-DBS stimulation, the ST group had a shorter *T*_1/2_ than the PD group (*P* = 0.0004), an increased *k*′ (*P* < 0.0001), a decreased D* (*P* = 0.0002), and a reduced *α* (*P* < 0.0001) (Fig. 2C to G). The effects of STN-DBS on ECS parameters across different groups were shown in Fig. 2 and Table 2.

**Table 2.** The effects of STN-DBS on ECS parameters across different groups

	D* (mm^2^/s)	*T*_1/2_ (min)	*k*′ (mm^2^/s)	*α* (%)
Sham group	3.09 ± 0.08	70.33 ± 9.66	3.11 ± 0.05	17.10 ± 0.08
PD group	4.21 ± 0.13	114.40 ± 7.16	2.28 ± 0.08	18.03 ± 0.15
NST group	4.12 ± 0.19	113.20 ± 6.01	2.38 ± 0.28	17.82 ± 0.22
ST group	3.74 ± 0.27	96.47 ± 5.89	2.82 ± 0.17	17.48 ± 0.17
*P*1	<0.0001	<0.0001	<0.0001	<0.0001
*P*2	<0.0001	<0.0001	<0.0001	<0.0001
*P*3	0.4247	0.7725	0.3623	0.0382
*P*4	0.0002	0.0004	<0.0001	<0.0001
*P*5	<0.0001	<0.0001	0.0089	0.0007

Data are presented as mean ± SD. Multiple group data comparisons were performed using one-way ANOVA with FDR correction. Statistical significance was set at *P* < 0.05. *P*1, Sham group versus PD group; *P*2, Sham group versus NST group; *P*3, NST group versus PD group; *P*4, ST group versus PD group; *P*5, Sham group versus ST group. PD, Parkinson’s disease; NST, electrode implantation but no stimulation; ST, electrode implantation and stimulation.

### Changes in EAAT2 expression and extracellular glutamate content in the impaired brain regions of PD rats after STN-DBS treatment

In comparison to the Sham group, the PD group exhibited a decreased EAAT2 ratio (*P* = 0.0019) and increased extracellular glutamate content (*P* < 0.0001). However, the ST group ameliorated this situation by increasing EAAT2 expression (*P* = 0.0003) and reducing extracellular glutamate levels (*P* = 0.0001) (Fig. 3B to D).

### Changes in HA in the impaired brain regions of PD rats after STN-DBS treatment

Using the ELISA kit and calculating the HA ratio (actual concentration/mean value), the results showed that, compared with the Sham group, the PD HA ratio was significantly decreased (*P* < 0.0001), while the ST group showed an increase in the HA content (*P* = 0.0001) (Fig. 3E).

### Expression of AQP-4 and GFAP in the impaired brain regions of PD rats after STN-DBS treatment

**Fig. 4.**
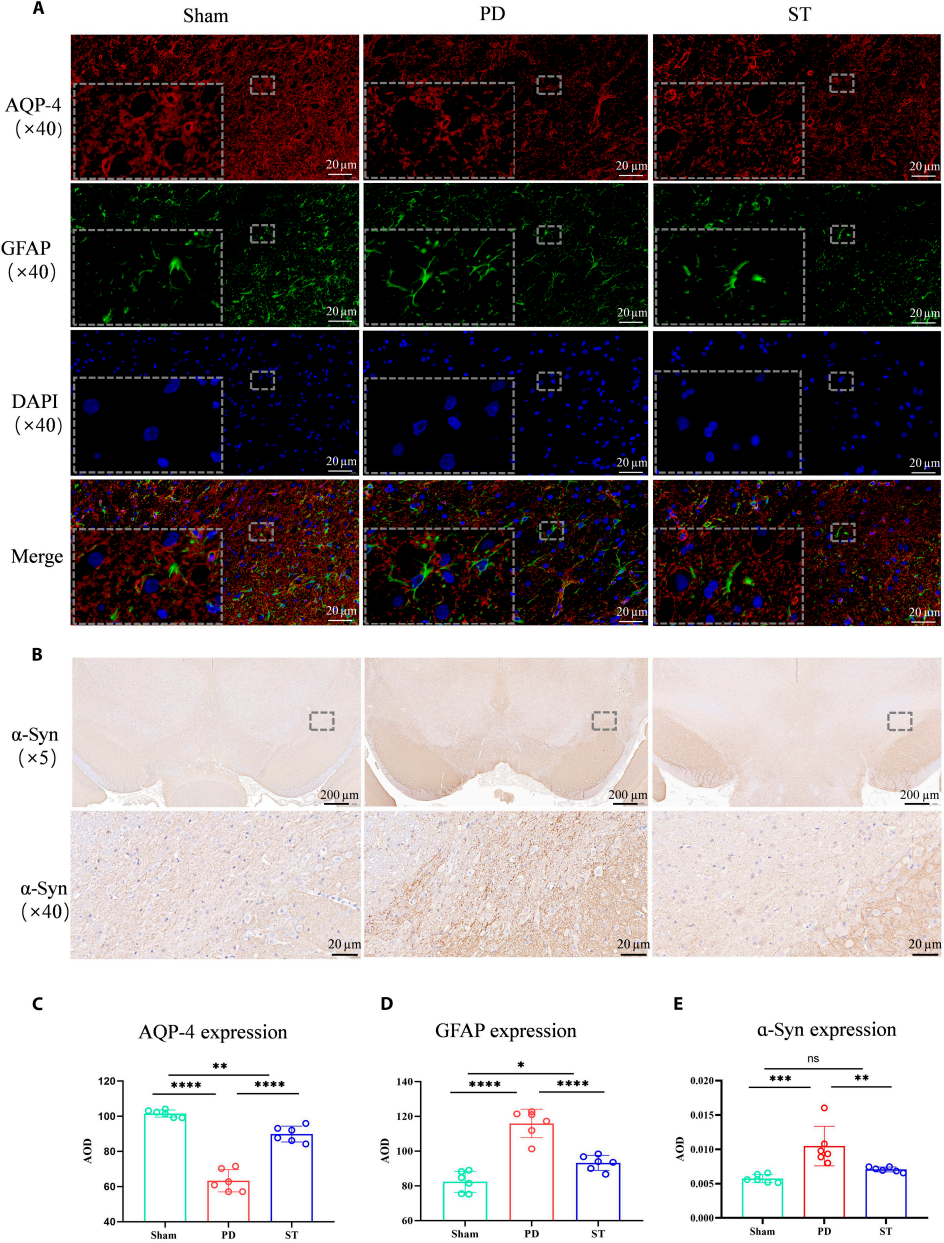
(A) Expression of AQP-4 and GFAP. Immunofluorescence findings indicate that in the right SN, the expression of AQP-4 (red) was reduced in the PD group relative to the Sham group, while the expression of GFAP (green) was elevated, evidenced by the thickening and elongation of astrocyte processes. Following treatment with STN-DBS, there was a partial recovery in AQP-4 expression in the ST group when compared to the PD group. Additionally, GFAP expression was reduced, and the morphology of astrocytes showed a trend toward normalization. (B) Immunohistochemical results for α-syn. Compared with the Sham group, extracellular α-syn expression increased significantly in the right SN of the PD group. After STN-DBS treatment, extracellular α-syn expression was reduced in the ST group. (C to E) Quantitative analysis of differences in AQP-4, GFAP, and α-syn expression between groups. The dashed white line represents the denser part of the SN. Circles represent individual values, and error bars represent SD. One-way ANOVA was performed followed by FDR correction. *P < 0.05, **P < 0.01, ***P < 0.001, ****P < 0.0001.

Immunofluorescence analysis disclosed that, relative to the Sham group, the average optical density (AOD) of AQP-4 was diminished in the PD group (*P* < 0.0001), whereas an increase was observed in the AOD of GFAP (*P* < 0.0001). It is noteworthy that the ST group augmented the expression of AQP-4 (*P* < 0.0001) and decreased GFAP expression (*P* < 0.0001) (Fig. 4A, C, and D).

### Changes in α-syn in the impaired brain regions of PD rats after STN-DBS treatment

Compared with the Sham group, α-syn in the injured SN region of the PD group increased significantly (*P* = 0.0002), while α-syn in the injured SN region of the ST group decreased (*P* = 0.0036) (Fig. 4B and E). The effects of STN-DBS on ECS molecular diffusion and ISF drainage across different groups were shown in Fig. 4 and Table 3.

**Table 3.** The effects of STN-DBS on ECS molecular diffusion and ISF across different groups

	WB	Microdialysis	ELISA	Immunofluorescence		Immunohistochemistry
	EAAT2	Glu	HA ratio	AQP-4	GFAP	α-Synuclein
Sham group	1.82 ± 0.54	0.27 ± 0.14	1.0 ± 0.098	101.5 ± 2.018	82.36 ± 6.037	57.34 ± 5.948
PD group	0.53 ± 0.32	2.31 ± 0.82	0.53 ± 0.089	63.3 ± 6.343	115.9 ± 8.18	104.7 ± 28.78
ST group	1.68 ± 0.50	0.75 ± 0.36	0.79 ± 0.065	89.85 ± 4.501	93.24 ± 4.303	70.65 ± 3.587
*P*1	0.004	<0.0001	<0.0001	<0.0001	<0.0001	0.001
*P*2	0.001	0	0.0001	<0.0001	<0.0001	0.009
*P*3	0.5607	0.1364	0.0007	0.001	0.0098	0.1973

Data are presented as mean ± SD. Multiple group data comparisons were performed using one-way ANOVA with FDR correction. Statistical significance was set at *P* < 0.05. *P*1, Sham group versus PD group; *P*2, PD versus ST; *P*3, Sham versus ST. PD, Parkinson’s disease; ST, electrode implantation and stimulation; ISF, interstitial fluid drainage; WB, Western blot; EAAT2, excitatory amino acid transporter 2; HA, hyaluronic acid; AQP-4, aquaporin-4; GFAP, glial fibrillary acidic protein.

### Correlation analysis results

**Fig. 5.**
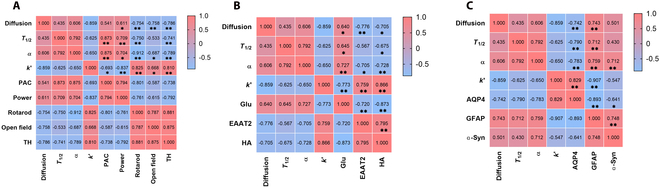
(A) Correlation analysis between ECS parameters and behavioral and EEG evaluation indices. (B) Correlation analysis between ECS parameters and EAAT2 expression, as well as glutamate and HA content. (C) Correlation analysis between ECS parameters and the expression of AQP-4, GFAP, and α-syn. *P < 0.05, **P < 0.01.

To further explore the correlation between neuroimaging indicators and microstructure, behavior, and EEG indicators, Pearson’s correlation analysis was performed (Fig. 5A to C).

*a* was positively correlated with PAC (Pearson correlation coefficient = 0.875, *P* = 0.000) and negatively correlated with TH (Pearson correlation coefficient = −0.789, *P* = 0.002).

D* was positively correlated with extracellular glutamate content (Pearson correlation coefficient = 0.640, *P* = 0.035) and negatively correlated with EAAT2, HA, AQP-4, and TH (Pearson correlation coefficients = −0.776, −0.705, −0.742, and −0.786, *P* = 0.003, 0.010, 0.005, and 0.002). *T*_1/2_ was negatively correlated with AQP-4 and TH levels (Pearson correlation coefficients = −0.790 and −0.741, *P* = 0.022 and 0.006). *k*′ was positively correlated with EAAT2, HA, AQP-4, and TH (Pearson correlation coefficients = 0.759, 0.866, 0.829, and 0.810, *P* = 0.004, 0.000, 0.001, and 0.001).

## Discussion

In this study, tracer-based MRI and Decs-mapping techniques were used to quantitatively measure the structural and functional characteristics of the ECS. The findings indicate that STN-DBS can regulate the content of HA in the ECS, promote the transport of excitatory neurotoxic substance glutamate, restore the expression of AQP-4 to accelerate ISF drainage, enhance the clearance of α-syn, maintain the homeostasis of the extracellular microenvironment, improve motor dysfunction, and protect dopaminergic neurons in the SN.

Accurate diagnosis is a prerequisite for achieving the optimal therapeutic effects of DBS in patients with PD. Currently, the diagnostic criteria for PD in guidelines worldwide primarily rely on medical history and physical signs [41]. Motor dysfunction is the primary clinical feature of PD [42]. Electrophysiological studies have revealed synchronized discharges and oscillations in the M1 and STN of patients with PD and nonhuman primates, which are associated with bradykinesia and stiffness [43,44]. Moreover, research indicates that the power spectrum of β oscillations and PAC strength in LFPs [45–47] can serve as key biomarkers for the detection and treatment evaluation of PD [48,49]. Our study combines the behavioral performance of experimental animals with β-band oscillations in the M1 area, providing a more objective and comprehensive evaluation of PD model establishment and DBS treatment efficacy. Our findings demonstrate that, compared with the Sham group, the average fall time of rats in the PD group was significantly shortened, the average movement speed was markedly reduced, and the average power intensity and PAC strength were enhanced, indicating the successful establishment of the PD model. After STN-DBS treatment, motor dysfunction was alleviated, with reductions in the average power intensity and PAC, which is consistent with the results of Hacker et al. [50] and Eusebio et al. [51], demonstrating the effectiveness of DBS in reducing the synchrony of oscillatory activity between the STN and the cortex and in treating motor dysfunction. To further demonstrate the protective effect of STN-DBS on dopaminergic neurons in the SN, immunohistochemical analysis of TH staining revealed an increase in the TH-immunopositive cell count in the STN-DBS group.

Previous evidence has indicated that dopaminergic neurons in the SN begin to undergo apoptosis before the onset of motor impairment. Motor impairments emerge only when the number of dopaminergic neurons decreases by over 80% [52]. Earlier research by our team revealed a close correlation between an increase in the *α* of brain ECS and the loss of dopaminergic neurons in the SN [34]. Owing to the larger cell bodies and longer neural fibers of dopaminergic neurons compared with those of other neural cells in the brain, they occupy more space. When a substantial number of dopaminergic neurons undergo apoptosis, the space occupied by the brain ECS increases markedly, leading to an increase in the volumetric fraction. Our study also reveals that, compared with the Sham group, the *α* of the damaged brain regions in rats with PD marked increased, while the number of TH-positive cells notably decreased, with a negative correlation between the 2. However, the findings of Reum et al. [53] differ from those of ours. They suggest a decrease in the *α* of PD rats with 6-OHDA-induced lesions, which they attribute to differences in the observed structures. Reum et al. studied the *α* of the striatum, where the ECS had already accumulated dopamine transmitted from the SN to the striatum [54], thereby occupying the ECS and leading to a reduction. This indicates that the *α* of the SN is an effective parameter reflecting the number of dopaminergic neurons and may aid in the early identification of PD. After treatment with STN-DBS, the *α* of the damaged brain regions in rats in the ST group was reduced, and the number of TH-positive cells increased, further confirming that STN-DBS can protect dopaminergic neurons in the SN and maintain the normal ECS structure of the brain.

The primary structural component of the brain ECS is the ECM, which is a dense network of high molecular weight components secreted by the glial cells and neurons [55,56]. As the main scaffold molecule, HA forms a physical barrier to molecular diffusion [57], and changes in its content affect ECS diffusion [58]. HA degradation may influence the phenotype and function of astrocytes through specific mechanisms [59]. What is more, changes in HA levels may regulate EAAT2 expression by affecting the integrity of the ECM and intercellular communication [60]. A decrease in its concentration is a important factor contributing to increased astrocyte reactivity and inflammatory responses. Neurons and astrocytes maintain the balance of neurotransmission in the central nervous system by regulating the glutamate/glutamine cycle [61]. Astrocytes are activated during the transport of excess glutamate, and the release of inflammatory factors may promote the release of glutamate into the ECS [62]. An increase in pro-inflammatory factor levels also leads to a decrease in the ability of astrocytes to transport glutamate, thereby creating a vicious cycle of increased inflammatory response and glutamate excitotoxicity. Research published by Mahmoud et al. indicates that excessive extracellular glutamate overstimulates muscular and metabolic glutamate receptors in the postsynaptic membrane, leading to neuronal excitotoxicity [63–65], which is one of the reasons for neuronal degeneration in PD [66]. The EAAT2 subtype present in astrocytes is functionally the primary regulator of extracellular glutamate levels in most brain regions [67] and can absorb 95% of the glutamate from the synaptic cleft [68]. Glutamate uptake is significantly reduced in patients with PD [69]. Earlier research by our team showed that Decs-mapping can be used to track neuronal activity deep in the brain, with D* serving as an indicator for assessing the excitatory neurotransmitter glutamate [70]. In our study, the PD group showed reduced HA content, increased D*, decreased EAAT2 expression, and increased extracellular glutamate levels in the SN. Correlation analysis revealed that HA was negatively correlated with *D* and glutamate and positively correlated with EAAT2, indicating that HA, as a physical barrier for molecular diffusion, plays a crucial role in the transport of extracellular glutamate. After STN-DBS treatment, the ST group showed an increase in HA content, a decrease in D*, an increase in EAAT2 expression, and a reduction in extracellular glutamate levels in the SN. Campos et al. [71] discovered that STN-DBS could also restore EAAT2 expression and promote the uptake of extracellular glutamate; however, their study did not consider the impact of DBS on the ECM. Our study complements previous studies by demonstrating that DBS can regulate HA in the ECM, repair the molecular barrier between the synaptic cleft and astrocytes, improve D*, restore EAAT2 expression, reduce glutamate excitotoxicity, and maintain homeostasis in the extracellular microenvironment.

ISF flow is the reason for the clearance of these intrabody delivery tracers [27,72]. *k*′ and *T*_1/2_ are parameters reflecting the ISF drainage velocity within the brain’s ECS [9,44]. An increasing number of researchers believe that impaired ISF drainage in the brain is a potential driving factor for neurodegeneration [73,74]. The imbalance between the production and clearance of harmful substances and their subsequent accumulation plays a crucial role in PD pathogenesis [23,75]. α-Syn is the most relevant harmful substance associated with the onset and progression of PD. The impaired clearance of cerebrospinal fluid α-syn reported by Parnetti et al. [76] can serve as a biomarker for the diagnosis of PD. AQP-4 is involved in ISF flow and drives the clearance of interstitial solutes from the brain parenchyma [20,77]. Recent research has revealed that abnormal AQP-4 expression is associated with PD. Studies by Xue et al. [78] revealed that, compared with wild-type controls, AQP-4-deficient mice are more sensitive to massive loss of TH-positive neurons and motor dysfunction. Other studies have analyzed AQP-4 expression in the SN and discovered that the density of AQP-4 around SNpc vessels in mice is higher than that in the cortex [79,80]. Therefore, the SN is more prone to water molecule accumulation, supporting the hypothesis that AQP-4 may be involved in the pathogenesis of PD. Cui et al. [81] discovered that reduced AQP-4 expression accelerated the pathological deposition of α-syn. Hamby and Sofroniew [82] discovered that changes in AQP-4 expression and localization in reactive astrocytes under neuropathological conditions may lead to disturbed ISF flow and failure to clear neurotoxic solutes such as α-syn. Our research revealed that, compared with the Sham group, *k*′ in the damaged SN region of the PD group was decreased, *T*_1/2_ was significantly increased, GFAP expression was increased, AQP-4 expression was decreased, and α-syn expression was increased. Correlation analysis revealed correlations between *k*′, *T*_1/2_, GFAP, and AQP-4, with *k*′ positively correlated with AQP-4 and negatively correlated with GFAP and *T*_1/2_, showing the opposite trends. This confirms that ECS parameters related to ISF drainage velocity (*k*′ and *T*_1/2_) can reflect AQP-4 expression and α-syn deposition. Following STN-DBS treatment, GFAP expression decreased, AQP-4 expression increased, and α-syn expression decreased. Campos et al. [83] found that STN-DBS partially reverses astrocyte hypertrophy and proliferation in a 6-OHDA-induced PD rat model, inhibiting astrocyte activation. This supports our results, demonstrating that DBS can regulate AQP-4 expression, improve ISF drainage, and reduce α-syn accumulation.

Additionally, the correlation analysis results indicated that among the indicators related to drainage (*T*_1/2_ and *k*′), *T*_1/2_ was positively correlated with power and PAC, while *k*′ was negatively correlated with power and PAC. This suggests that a certain correlation exists between β-band power and PAC and ISF drainage. In our study, the emergence of β-band in the PD group rats disrupted the rhythmic oscillation of neurons, hindering ISF drainage and waste clearance. This is consistent with the findings presented by Jiang-Xie et al. [84], which revealed that rhythmic neuronal oscillations influence ISF drainage and the clearance of brain waste. In our study, STN-DBS restored neuronal rhythmic oscillations and improved drainage impairments, further confirming that STN-DBS accelerated ISF drainage and waste clearance in the ECS of PD rats.

Although the NST group also experienced alleviation of motor dysfunction, the number of TH-immunopositive cells in the right SN did not increase, and there were no statistically significant differences in ECS parameters between the NST group and the PD group. This may be related to microlesion effects in the STN. The microlesion effect [85] refers to the minute damage to the brain tissue caused by electrode implantation during DBS surgery, which may contribute to symptom relief. Analysis of our research results suggests that this phenomenon is not achieved through the protection of neuronal structures but may be attributed to rapid onset mechanisms at the electrophysiological level, such as stimulation of the hyperdirect pathway in the STN-M1 region after electrode implantation [86,87], which can retrogradely activate motor cortex projection neurons to generate retrograde action potentials and inhibit cortical β synchronization oscillations, thereby improving motor dysfunction in PD.

### Limitations and future work

In our study, we have elucidated the mechanisms by which DBS alleviates PD symptoms through ECS modulation, offering novel insights into therapeutic approaches. However, it is crucial to recognize the study’s limitations. First, our use of the 6-OHDA model to mimic dopaminergic neuron degeneration and associated motor deficits captures only certain aspects of human PD, omitting the disease’s genetic, environmental, and neuroinflammatory complexities. Second, although MRI provided a noninvasive 3D evaluation of ECS dynamics, it was subject to limitations such as uneven probe distribution and limited resolution. Third, our focus was on ECS parameter changes before and after DBS treatment, and we investigated ECS modulation under specific DBS frequencies and disease stages to reflect clinical practice.

Future research is warranted to translate these findings to human studies. First, to more accurately reflect human PD pathology, it is crucial to utilize diverse animal models, including transgenic rodents and those with chronic dopaminergic lesions. Second, designing clinical trials with clear objectives and appropriate participant criteria is essential to investigate ECS alternations following STN-DBS in humans. Third, the application of advanced technologies, such as implantable sensors, functional MRI, and optical imaging, could enable continuous tracking of ECS changes over time.

### Conclusion

In this study, we conducted a thorough investigation into the modifications of ECS parameters in a rat model of PD, both preceding and subsequent to STN-DBS intervention. Our results compellingly demonstrate that STN-DBS exerts its therapeutic efficacy by regulating extracellular ECM constituents and enhancing ISF drainage, thereby improving the extracellular microenvironment and preserving dopaminergic neurons. These findings not only contribute to a deeper understanding of the mechanisms underlying STN-DBS in PD treatment but also provide a robust theoretical framework for its application. Furthermore, they open up new avenues for the exploration of therapeutic targets and the optimization of clinical protocols, holding promise for the development of more efficacious treatment strategies in the future.

## Data Availability

The data are contained within the article and the Supplementary Materials.
