# Supplementary material for: Remote Regulation of Molecular Diffusion in Extracellular Space of Parkinson’s Disease Rat Model by Subthalamic Nucleus Deep Brain Stimulation
Source: Cyborg Bionic Syst. 2025 Apr 3;6:0218. doi: 10.34133/cbsystems.0218 (PMC11969791; doi:10.34133/cbsystems.0218)
Supplement: Supplementary 1 — Fig. S1 [file cbsystems.0218.f1.docx]

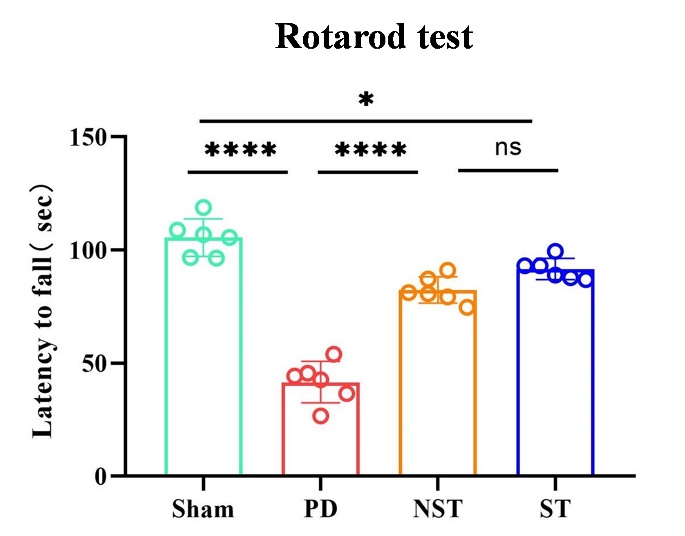


**Supplement Fig.1** Results of the Rotarod test indicate that compared to the Sham group (n=6), the average locomotor speed of rats in the PD group (n=6) was significantly reduced. The NST group (n=6) and the ST group (n=6) showed improved average locomotor speed compared to the PD group, and there was no statistical difference between the NST group and the ST group. **p < 0.01; ****p < 0. 0001; ns, not significant. One-way ANOVA with Tukey's multiple comparisons test.
